# Supplementary material for: Seminoma and Embryonal Carcinoma Footprints Identified by Analysis of Integrated Genome-Wide Epigenetic and Expression Profiles of Germ Cell Cancer Cell Lines
Source: PLoS One. 2014 Jun 2;9(6):e98330. doi: 10.1371/journal.pone.0098330 (PMC4041891; doi:10.1371/journal.pone.0098330)
Supplement: File S1 — ZIP file containing DMRforPairs output for significant regions. Please start from the html files. (ZIP) [file pone.0098330.s008.zip › figures/1298.pdf]

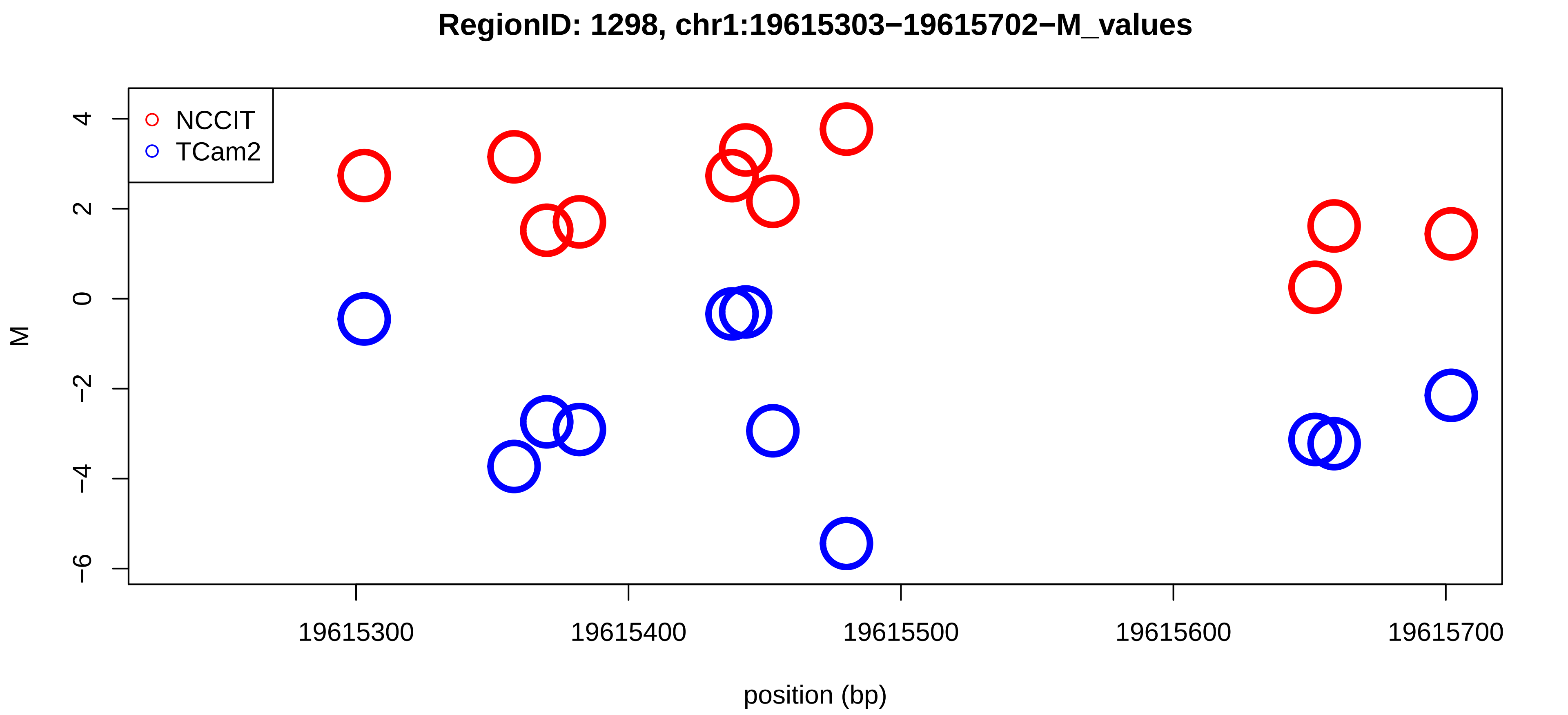

RegionID: 1298, chr1:19615303−19615702−M_values
NCCIT
TCam2
M
4
2
0
−2
−4
−6
19615300
19615400
19615500
19615600
19615700
position (bp)

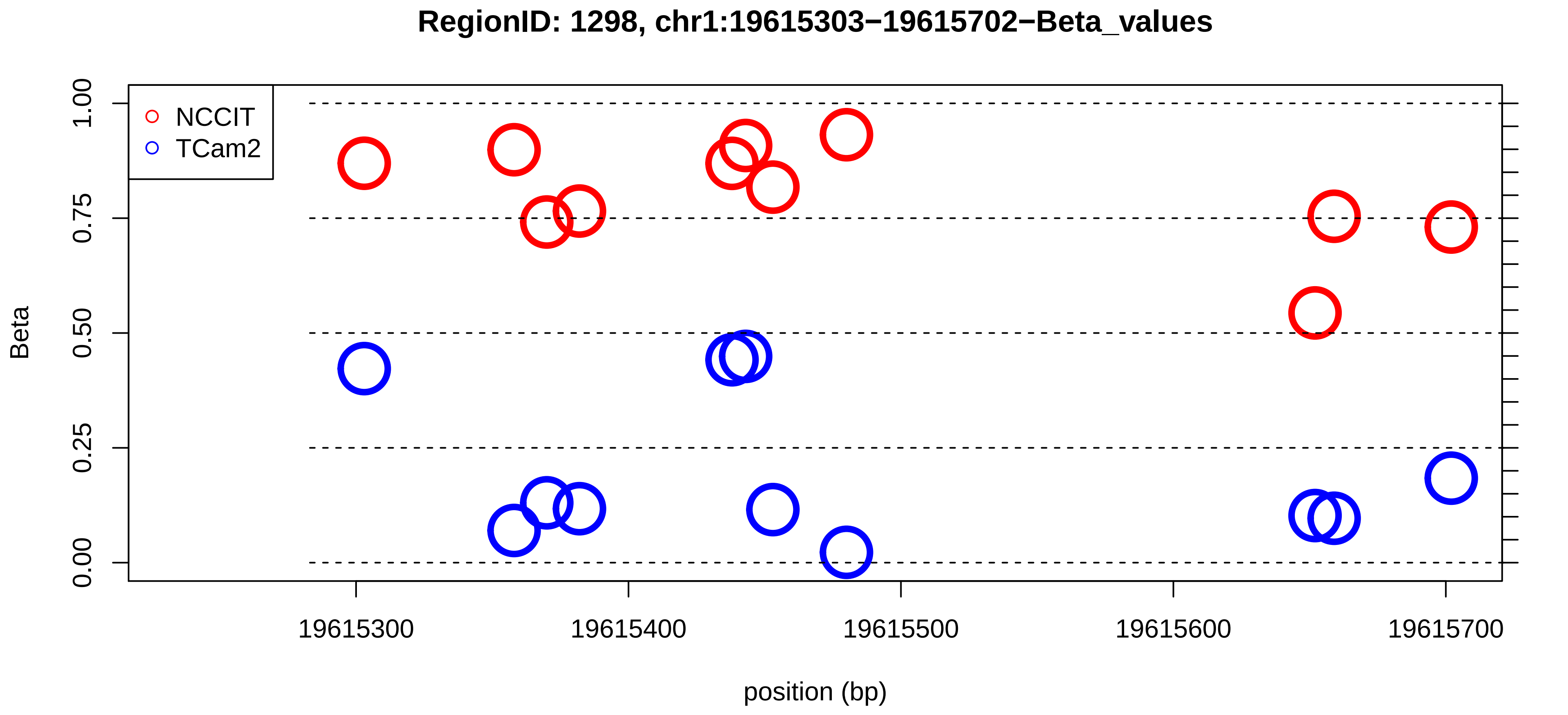

RegionID: 1298, chr1:19615303−19615702−Beta_values
NCCIT
TCam2
Beta
1.00
0.75
0.50
0.25
0.00
19615300
19615400
19615500
19615600
19615700
position (bp)

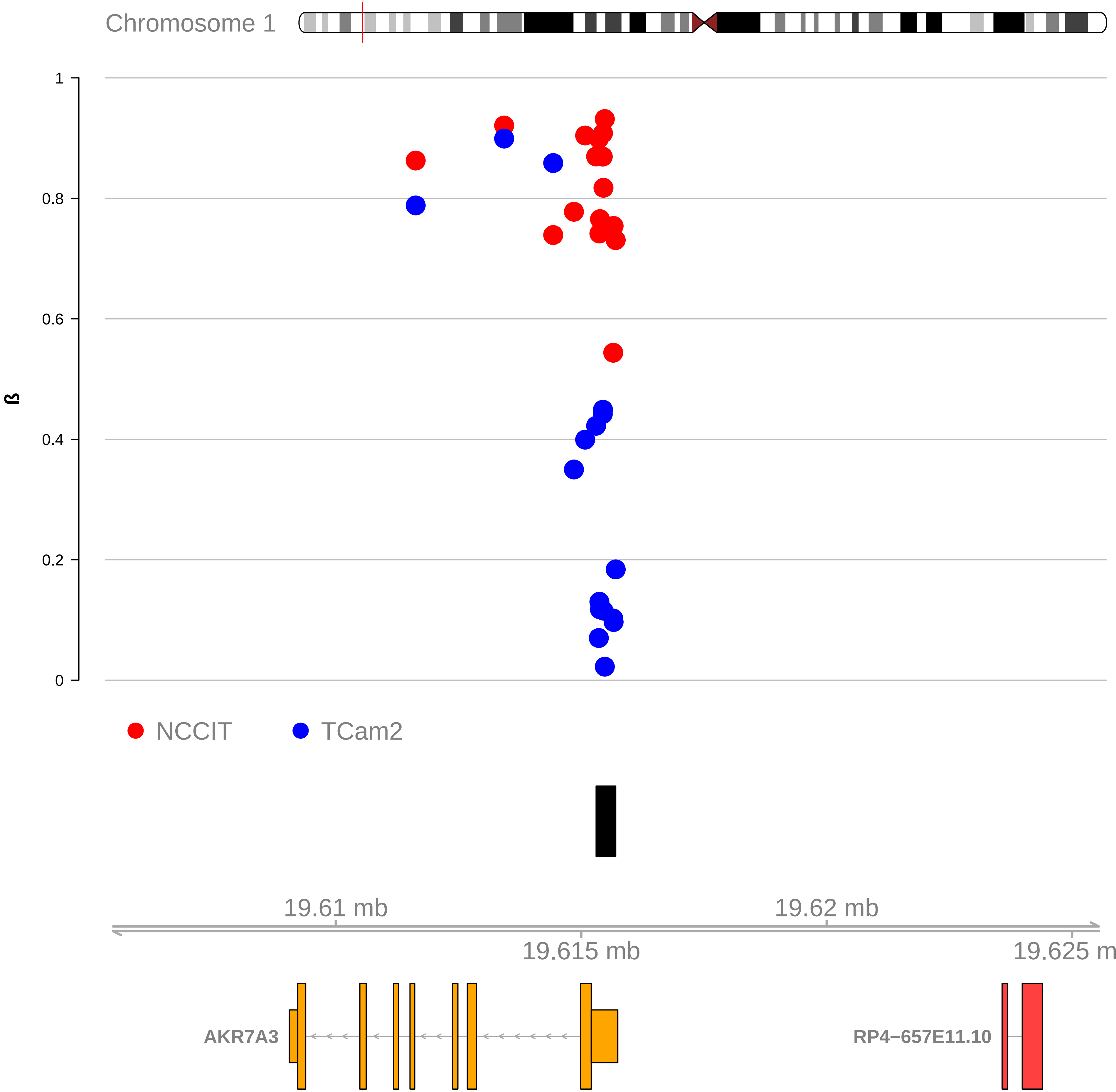

Chromosome 1
1
0.8
0.6
0.4
0.2
0
ß
NCCIT
TCam2
19.61 mb
19.62 mb
19.615 mb
19.625 m
AKR7A3
RP4−657E11.10
